# Characterization data of gilthead sea bream (*Sparus aurata*) IGF-I receptors (IGF-IRa/Rb)

**DOI:** 10.1016/j.dib.2015.12.046

**Published:** 2016-01-06

**Authors:** Emilio J. Vélez, Sheida Azizi, Cristina Salmerón, Shu Jin Chan, Mohammad Ali Nematollahi, Bagher Mojazi Amiri, Isabel Navarro, Encarnación Capilla, Joaquim Gutiérrez

**Affiliations:** a Departament de Fisiologia i Immunologia, Facultat de Biologia, Universitat de Barcelona, Avinguda Diagonal 643, 08028 Barcelona, Spain; b Department of Biochemistry and Molecular Biology and Department of Medicine and the Howard Hughes Medical Institute, University of Chicago, Chicago, IL 60637, USA; c Department of Fisheries Sciences, Faculty of Natural Resources, University of Tehran, Karaj, Iran; d Departament de Fisiologia i Immunologia, Facultat de Biologia, Universitat de Barcelona, 08028 Barcelona, Spain. Visitor PhD student

**Keywords:** IGF-I receptors, Gilthead sea bream

## Abstract

In this data article we describe the coding sequence of two IGF-IR paralogues (IGF-IRa and IGF-IRb) obtained from gilthead sea bream embryos. The putative protein architecture (domains and other important motifs) was determined and, amino acid sequences alignment and phylogenetic analysis of both receptors together with IGF-IR orthologues from different vertebrates was performed. Additionally, a semi-quantitative conventional PCR was done to analyze the mRNA expression of both receptors in different tissues of gilthead sea bream. These data will assist in further physiological studies in this species. In this sense, the expression of both receptors during ontogeny in muscle as well as the differential effects of IGF-I and IGF-II on their regulation during *in vitro* myogenesis has been recently studied (doi: 10.1016/j.ygcen.2015.11.011; [1]).

**Specifications Table**

**Table:** 

Subject area	*Biology*
More specific subject area	*Fish endocrinology*
Type of data	*Tables and figures*
How data was acquired	*Conventional and RACE PCR in a BioRad thermocycler*
Data format	*Analyzed*
Experimental factors	*Samples were extracted in sterile conditions and then quickly frozen in liquid nitrogen.*
Experimental features	*Total RNA from embryos and different tissues of gilthead sea bream was extracted using TRI Reagent Solution (Applied Biosystems). RNA quantity and quality was analyzed by a NanoDrop 2000 (Thermo Scientific). cDNA synthesized using the Affinity Script*^*TM*^*QPCR cDNA Synthesis Kit (Agilent Technologies). PCRs run in a BioRad thermocycler and sequencing performed using the BigDye Terminator v3.1 Cycle Sequencing Kit (Applied Biosystems) by the scientific services of the University of Barcelona.*
Data source location	*University of Barcelona, Barcelona, Spain*
Data accessibility	*Data in this article is deposited in GenBank (NCBI) under accession numbers: GenBank: KT156846 for IGF-IRa and GenBank: KT156847 for IGF-IRb.*

**Value of the data**

• It provides the sequences and characterization of two IGF-I receptors in gilthead sea bream (*Sparus aurata*).
• These data open the opportunity to study the GH/IGF-I axis in this species.
• It facilitates scientists to further physiological studies in gilthead sea bream.

## 1 Data

**Fig. 1:**
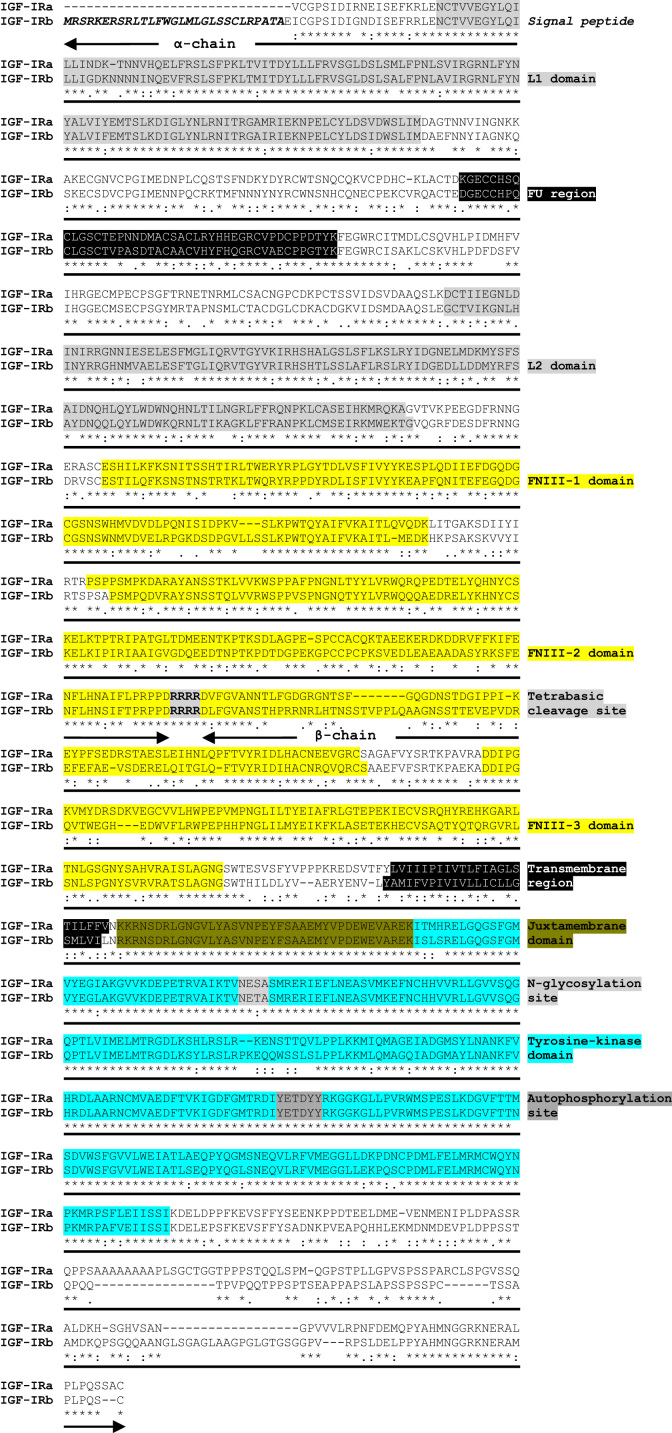
Comparison of gilthead sea bream IGF-IRa and IGF-IRb protein sequences. Alignment of deduced amino acid sequences of both gilthead sea bream IGF-IRs via MAFFT and G-INS-i method. Symbols: (*) identical residues in both sequences; (:) conservative substitutions and (.) semiconservative substitutions. The putative molecular architecture was determined according to NCBI and SMART tools. All important domains or amino acid motifs are indicated. L: leucine-rich; FU: furin-like; FNIII: fibronectin type 3.

**Fig. 2:**
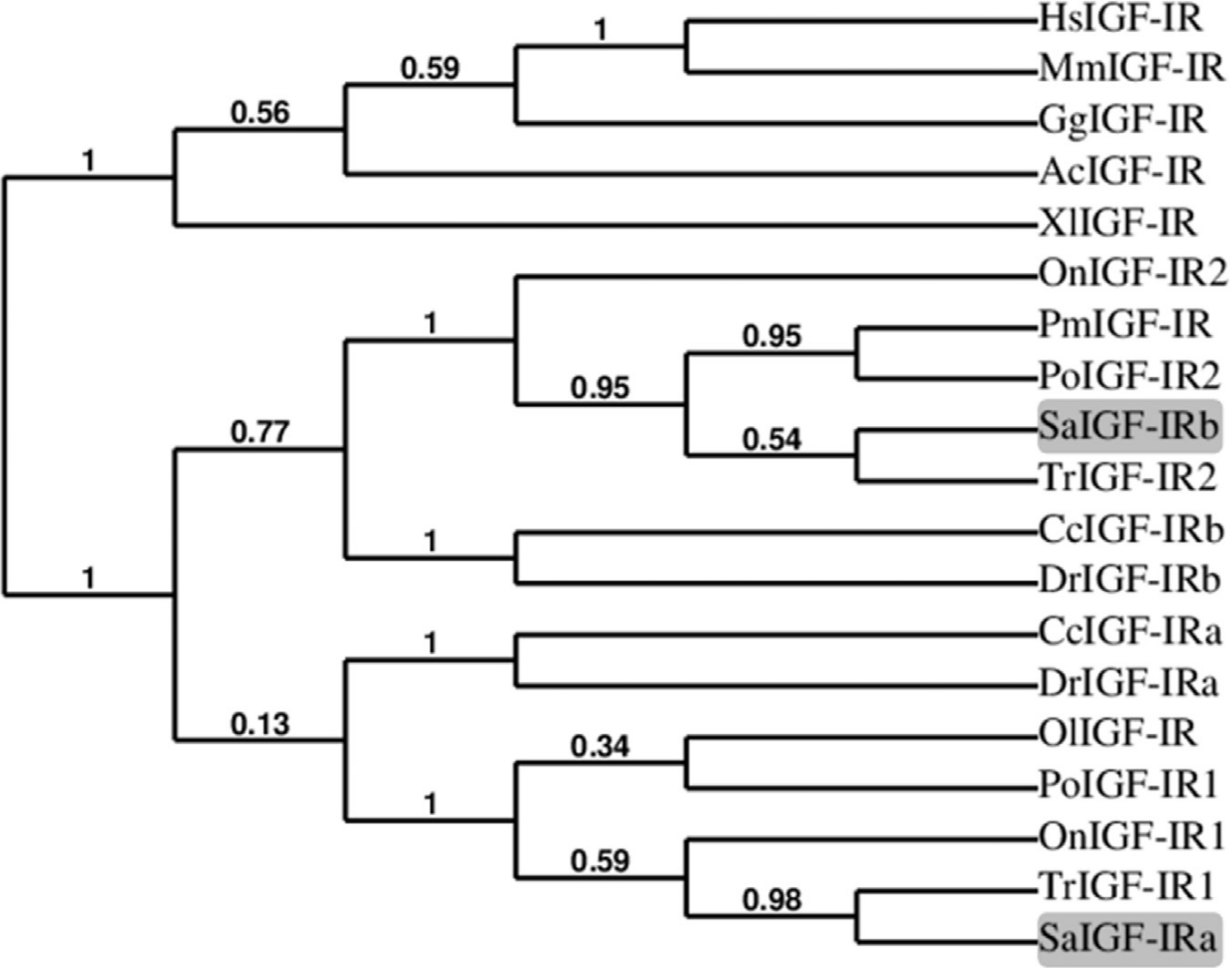
Phylogenetic analysis of IGF-IRs. Unrooted tree predicting the evolutionary relationship between *Sparus aurata* (Sa), *Anolis carolinensis* (Ac), *Cyprinus carpio* (Cc), *Danio rerio* (Dr), *Gallus gallus* (Gg), *Homo sapiens* (Hs), *Mus musculus* (Mm), *Oreochromis niloticus* (On), *Oryzias latipes* (Ol), *Paralichthys olivaceus* (Po), *Psetta maxima* (Pm), *Takifugu rubripes* (Tr) and *Xenopus laevis* (Xl) IGF-IRs orthologues was created using PhyML. Bootstrap values are indicated at the nodes.

**Fig. 3:**
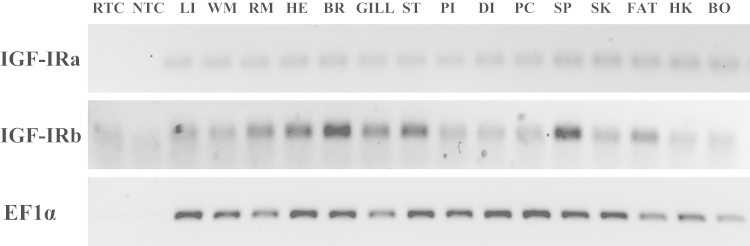
Tissue distribution of gilthead sea bream IGF-IRa and IGF-IRb. RTC: No Reverse Transcriptase Control, NTC: No Template Control, LI: liver, WM: white muscle, RM: red muscle, HE: heart, BR: brain, GILL, ST: stomach, PI: proximal intestine, DI: distal intestine, PC: pyloric caeca, SP: spleen, SK: skin, FAT: adipose tissue, HK: head kidney, BO: bone. A representative experiment from *n*=3 independent fish analyzed is shown.

Two coding sequences were obtained for IGF-IRs in gilthead sea bream and deposited in GenBank as IGF-IRa (Accession number: KT156846) and IGF-IRb (Accession number: KT156847). The sequence analysis revealed that both receptors are composed by one α and one β subunits connected by the characteristic tetrabasic cleavage site and, are organized into several major domains characteristic of this family of receptors (Fig. 1). The phylogenetic analysis showed that the tree divides first into two distinct branches, one including the mammalian, avian, reptilian and amphibian sequences and, a second one containing only fish orthologues, which separates again creating two clusters containing one paralogue each (Fig. 2). Furthermore, the two gilthead sea bream IGF-IRs shared a protein identity of 69% and had values of comparison with other teleosts that ranged among 62–86% (Table 1). Additionally, a semi-quantitative PCR analysis of both receptors showed that IGF-IRa was expressed equally in all tissues analyzed, whereas IGF-IRb presented differential expression between tissues, being more expressed in brain, spleen, stomach, heart and gill, and to a lower extent in the remaining tissues (Fig. 3).

## 2 Experimental design, material and methods

### 2.1 RNA extraction and cDNA synthesis

Total RNA was extracted from embryos or tissues (40–500 mg depending on tissue yield) with 1 mL of TRI Reagent Solution (Applied Biosystems, Alcobendas, Spain) following the manufacturer׳s instructions. Using a NanoDrop2000 (Thermo Scientific, Alcobendas, Spain) total RNA concentration and purity were determined. The integrity of the different samples was confirmed in a 1% agarose gel (w/v) stained with SYBR-Safe DNA Gel Stain (Life Technologies, Alcobendas, Spain). After that, 1 µg of total RNA was treated with DNase I (Life Technologies, Alcobendas, Spain) to remove all genomic DNA, and then reverse transcribed (RT) with the Affinity ScriptTM QPCR cDNA Synthesis Kit (Agilent Technologies, Las Rozas, Spain) following the manufacturer’s recommendations.

### 2.2 Cloning and sequencing

The sequences of two gilthead sea bream IGF-IRs (IGF-IRa and IGF-IRb) were obtained from embryos using RT-PCR coupled to RACE methodology using a 5′ and 3′ RACE System for Rapid Amplification of cDNA Ends Kit as previously related [2]. First, degenerated primers against human IGF-IR (Accession number: BC143721) previously reported [3] were used and then, specific primers were designed using the DNAMAN software package (Lynnon Corporation, Quebec, Canada) and Net primer (http://www.premierbiosoft.com/netprimer/ Premier BioSoft, Palo Alto, CA, USA). After gel electrophoresis, the PCR products obtained were purified using a PureLink Quick Gel Extraction Kit, ligated into T/A pCR4-TOPO vector and transformed by thermal shock into TOP10 *Escherichia coli* cells (all from Invitrogen, Alcobendas, Spain). Later, several clones of each PCR product were sequenced using BigDye Terminator v3.1 Cycle Sequencing Kit (Applied Biosystems, Alcobendas, Spain) and finally, using DNAMAN the sequenced products were assembled into contigs with a final single coding sequence. Sequences generated were analyzed for similarity with other known sequences using the BLAST programs.

### 2.3 Sequence and phylogenetic analyses

The putative protein architecture of the IGF-IRs sequences (domains and other important motifs) was determined according to the literature and the conserved domain search program of NCBI [4] and the simple modular architecture research tool SMART version 4.0 (http://smart.embl-heidelberg.de) [5]. All the alignments were created with MAFFT version 7.220 (http://mafft.cbrc.jp/alignment/software/) and G-INS-i (recommended for <200 sequences with global homology) strategy. Sequences used other than those cloned in the present data set were obtained from NCBI. The phylogenetic tree was constructed using Phylogeny.fr (www.phylogeny.fr) [6] based on the phylogenetic estimation using the Maximum Likelihood (PhyML) software version 3.1/3.0 aLRT and MUSCLE version 3.8.31 [7] for the multiple sequence alignments.

### 2.4 Tissue screening

The IGF-IRa and IGF-IRb gene expression from 3 individual fish of 151±12 g on average was analyzed by qualitative RT-PCR using elongation factor 1-alpha (EF1α) as a control loading gene as related by [2]. Primers details are given in Table 2. Each reaction product was separated by agarose gel electrophoresis and visualized using SYBR Safe DNA gel stain (Life Technologies, Alcobendas, Spain) in a LAS-3000 (Fujifilm, Madrid, Spain) to confirm that a single product was amplified.

## Figures and Tables

**Table 1:** Percentages of sequence identity of IGF-IRs. Percentages of amino acid sequence identity between the *Sparus aurata* IGF-IRa and IGF-IRb and *Anolis carolinensis*, *Cyprinus carpio*, *Danio rerio*, *Gallus gallus*, *Homo sapiens*, *Mus musculus*, *Oreochromis niloticus*, *Oryzias latipes*, *Paralichthys olivaceus*, *Psetta maxima*, *Takifugu rubripes* and *Xenopus laevis* IGF-IRs orthologues.

**Species**	**Type**	**Accession no.**	**Protein identity (%) IGF-IRa/b**
*Anolis carolinensis*	IGF-IR	XM_003226491	64/64
*Cyprinus carpio*	IGF-IRa	AY144591	75/72
	IGF-IRb	AY144592	70/70
*Danio rerio*	IGF-IRa	NM_152968	74/72
	IGF-IRb	BC163581	69/70
*Gallus gallus*	IGF-IR	NM_205032	65/64
*Homo sapiens*	IGF-IR	BC143721	65/64
*Mus musculus*	IGF-IR	XM_006540643	62/63
*Oreochromis niloticus*	IGF-IR1	XM_003440598	82/68
	IGF-IR2	XM_005447691	66/82
*Oryzias latipes*	IGF-IR	XM_004069661	78/66
*Paralichthys olivaceus*	IGF-IR1	AB065098	84/69
	IGF-IR2	AB065099	66/83
*Psetta maxima*	IGF-IR	AJ224993	66/82
*Takifugu rubripes*	IGF-IR1	XM_003967057	83/67
	IGF-IR2	XM_003969432	66/86
*Xenopus laevis*	IGF-IR	NM_001088265	62/62

**Table 2:** Primers used in the PCR analyses. F: forward; R: reverse; Ta: annealing temperature.

**Gene**	**Primer sequences (5′–3′)**	**Ta (°C)**	**Accession No.**	**References**
*EF1a*	F: CTTCAACGCTCAGGTCATCAT	60	AF184170	8
R: GCACAGCGAAACGACCAAGGGGA
*IGF-IRa*	F: AGCATCAAAGACGAACTGG	55	KT156846	7
R: CTCCTCGCTGTAGAAGAAGC
*IGF-IRb*	F: GCTAATGCGAATGTGTTGG	55	KT156847	7
R: CGTCCTTTATGCTGCTGATG
