# Supplementary material for: Characterization data of gilthead sea bream (Sparus aurata) IGF-I receptors (IGF-IRa/Rb)
Source: Data Brief. 2016 Jan 6;6:507–13. doi: 10.1016/j.dib.2015.12.046 (PMC4724695; doi:10.1016/j.dib.2015.12.046)
Supplement: Supplementary file 1 — Supplementary material [file mmc1.doc]

Barcelona, 9^th^ December 2015

Dear Editor,

On behalf of all the authors, I declare that we do not have any conflict of interest.

Sincerely yours,

Joaquim Gutiérrez
